# The potential role of ChatGPT and artificial intelligence in anatomy education: a conversation with ChatGPT

**DOI:** 10.1007/s00276-023-03229-1

**Published:** 2023-08-16

**Authors:** Trifon Totlis, Konstantinos Natsis, Dimitrios Filos, Vasilios Ediaroglou, Nikolaos Mantzou, Fabrice Duparc, Maria Piagkou

**Affiliations:** 1 Department of Anatomy and Surgical Anatomy, School of Medicine, Faculty of Health Sciences, Aristotle University of Thessaloniki, 54124 Thessaloniki, Greece; 2 Laboratory of Computing, Medical Informatics and Biomedical-Imaging Technologies, School of Medicine, Aristotle University of Thessaloniki, Thessaloniki, Greece; 3 Department of Anatomy, Faculty of Medicine-Pharmacy, University of Rouen-Normandy, 22 Boulevard Gambetta, 76183 Rouen, France; 4 Department of Anatomy, School of Medicine, Faculty of Health Sciences, National and Kapodistrian University of Athens, Athens, Greece

**Keywords:** Anatomy education, Medical education, Artificial intelligence, Generative language models, ChatGPT

## Abstract

**Purpose:**

A recent study published in the JMIR Med Educ Journal explored the potential impact of the Generative Pre-Train (ChatGPT), a generative language model, on medical education, research, and practice. In the present study, an interview with ChatGPT was conducted to determine its capabilities and potential for use in anatomy education (AE) and anatomy research (AR).

**Methods:**

The study involved 18 questions asked of ChatGPT after obtaining an online subscription to the 4th edition. The questions were randomly selected and evaluated based on accuracy, relevance, and comprehensiveness.

**Results:**

The ChatGPT provided accurate and well-structured anatomical descriptions, including clinical relevance and relationships between structures. The chatbot also offered concise summaries of chapters and helpful advice on anatomical terminology, even with complex terms. However, when it came to anatomical variants and their clinical significance, the chatbot’s replies were inadequate unless variants were systematically classified into types. ChatGPT-4 generated multiple-choice quizzes and matching questions of varying difficulty levels, as well as summaries of articles when presented with text. However, the chatbot recognized its limitations in terms of accuracy, as did the authors of the current study.

**Conclusion:**

ChatGPT-4 can be a valuable interactive educational tool for students in the field of anatomy, encouraging engagement and further questions. However, it cannot replace the critical role of educators and should be used as a complementary tool. Future research should establish guidelines for ChatGPT’s optimal use and application in medical education.

## Introduction

For several years, consumers have had access to chatbots and conversational agents thanks to the rapid advancement in technology, specifically in the field of Artificial Intelligence (AI). In 2018, Open AI developed a language model called Generative Pre-Train (GPT), which eventually led to the creation of ChatGPT-3.5 in November 2022. This chatbot was unlike any other, boasting approximately 175 billion parameters and 570 GB of data, enabling it to discuss a wide range of topics. In just its first month of release, ChatGPT-3.5 garnered a whopping 57 million users and by January 2023, had reached 100 million users, making it the fastest-growing consumer application of all time.

In the Anatomical Sciences Education Journal, a letter to the editor was published that presented a conversation between the author and ChatGPT-3.5 [2]. The conversation included six questions related to anatomical facts, and it was found that ChatGPT did not consistently provide accurate information. Additionally, a study by Eysenbach in JMIR Med Educ [1] examined the potential of ChatGPT in medical education, research, and practice (ME, MR, and MP).

The current study involved an unedited interview with ChatGPT-4 to investigate its current abilities and potential for anatomy education (AE) and anatomy research (AR).

## Methods

After an online subscription to ChatGPT (4th version, edition of 24th of May), several questions regarding its potential role for AE and AR were asked of the chatbot. The questions flow was based on the reply to our first question about the several roles that the generative language models can play in AE. Questions topics were randomly selected after a consensus between the authors. Further questions were included to investigate the ChatGPT’s ability to address any issues related to anatomical variants and clinical and applied anatomy. All replies have not been copyedited or altered. Three authors (TT, KN, and MP), and professors of anatomy evaluated the ChatGPT’s replies in terms of accuracy, relevance to the question, and comprehensiveness.

## Results—the interview with the ChatGPT

### Interviewers (IVS)

What is the role of generative language models in medical students’ AE?

*ChatGPT*: Generative language models like GPT-4 have the potential to revolutionize ME in many ways, including AE. Here are several roles they can play:

1. *Detailed explanations*: Generative models can provide explanations on different parts of the human anatomy, their functions, and interconnections, making the subject more understandable and engaging.
2. *Question-replying*: Students can ask detailed or specific questions about anatomy, and these models can generate replies based on the vast amount of data they have been trained on. This allows students to learn at their own pace, revisit concepts, and explore areas of specific interest.
3. *Interactive learning*: With these models, is possible to create interactive learning experiences. For example, a virtual conversation with a “virtual doctor” or a “virtual patient” can be arranged to allow students to practice their diagnostic or communication skills.
4. *Simulated scenarios*: Generative models can be used to generate simulated medical cases, where students can practice diagnosing based on symptoms, patient history, and anatomy knowledge. This practical application can reinforce their learning and prepare them for real-life situations.
5. *Study guides and summaries*: The AI can create study guides or summaries based on a student’s needs, focusing on particular areas of anatomy or summarizing key concepts for revision.
6. *Language translation*: Generative models could help to break down language barriers in ME, translate complex anatomy content into different languages, and allow students from various linguistic backgrounds to learn effectively.
7. *Continuous updates*: AI models can be updated with the latest research findings, offering the most current knowledge on human anatomy, though it’s essential to note that, as of my last update in September 2021, this update would need to be done manually by retraining the model. While these are promising uses, it is also crucial to consider the limitations and ethical implications of using AI in education. For instance, the accuracy of AI replies should be validated, privacy should be respected, and the use of AI should complement but not replace the critical role of human educators.

### IVS

The first point is “Generative models can provide explanations on different parts of the human anatomy, their functions, and their interconnections, making the subject more understandable and engaging”. Can you explain how muscle origin and insertion are defined?

ChatGPT; Sure, let’s talk about muscle origins and insertions.

*Muscle origin:* This is typically the fixed, immovable, or less movable part of the muscle that is attached to the bone. The origin is usually proximal, meaning it’s closer to the center of the body. The origin point serves as the anchor of the muscle; when the muscle contracts, it pulls towards the origin. For example, in the case of the biceps brachii (BB) in the arm, the origins are at the scapula, which remains relatively stationary when the BB contracts. 

*Muscle insertion*: This is the movable attachment point of the muscle, usually located on the bone that the muscle moves when it contracts. The insertion is usually distal, meaning it’s further from the center of the body. When a muscle contracts, the insertion point is pulled towards the origin point, causing movement. In the example of the BB, the insertion is at the radius, which is moved when the BB contracts to flex the elbow. The origin of a muscle is the part that stays put during contraction, while the insertion is the part that moves. This principle is fundamental for understanding how muscles work to facilitate bodily movements.

### IVS

Let’s talk about your second point, “Students can ask detailed or specific questions about anatomy, and these models can generate responses”. Which are the branches of the axillary artery (AA)?

ChatGPT; The AA, a major blood vessel of the upper limb, has several branches that supply various structures in the shoulder and upper limb. After the subclavian artery exits the first rib, it becomes the AA. The AA is traditionally divided into three parts, with each part having specific branches:

- *1st part*: Is located between the outer border of the first rib and the medial border of the pectoralis minor muscle (Pm). It has one branch, the superior thoracic artery (STA) which supplies the chest wall and shoulder upper part.
- *2nd part*: Is located posterior to the Pm. It has two branches: 1. the thoracoacromial artery (TAA) with multiple branches supplying the pectoralis muscles, acromion, clavicle, and surrounding areas, and 2. The lateral thoracic artery (LTA) supplies the lateral chest wall and part of the mammary gland.
- *3rd part*: Extends from the lateral border of the Pm to the lower border of the teres major muscle. It has three branches: 1. The subscapular artery (SSA): the largest branch, that further divides into the thoracodorsal artery (TDA) and the circumflex scapular artery (CSA). It supplies parts of the shoulder and the upper back. 2. The anterior circumflex humeral artery (ACHA) circles the humerus, supplying the shoulder joint and nearby muscles. 3. The posterior circumflex humeral artery (PCHA) also circles the humerus but on the posterior side. Remember that variants can occur, and the exact AA branching pattern can sometimes vary from person to person.

### IVS

In the same way, could you provide a study guide for the “anatomy of the stomach”?

ChatGPT; Absolutely, here’s a brief study guide covering the major anatomical points of the stomach.

1. *Location*: In the upper left quadrant of the abdomen, just beneath the diaphragm. It is surrounded by the liver to its right, the spleen to its left, and the pancreas to its inferior.
2. *Parts of the stomach*: a. Cardia: the upper, narrow part of the stomach around the opening where the esophagus enters the stomach, b. Fundus: the rounded part of the stomach to the left of the cardia, beneath the diaphragm, c. Body (corpus): the main, central region of the stomach, d. Antrum: the lower part of the stomach, where food mixes with gastric juices to form chyme, e. Pylorus: the very bottom of the stomach that connects to the duodenum (the first part of the small intestine). It contains the pyloric sphincter, which regulates the flow of chyme into the duodenum.
3. *Layers of the stomach wall*: a. Mucosa: the innermost layer. It is composed of epithelium (which contains gastric pits leading to gastric glands), lamina propria, and muscularis mucosa, b. Submucosa: contains connective tissue, blood vessels, and nerves, c. Muscularis externa: composed of three layers of smooth muscle (an inner oblique, a middle circular, and an outer longitudinal layer) that help mix and churn the food, d. Serosa: the outermost layer, which is the peritoneum continuation.
4. *Gastric secretions*: The stomach secretes a variety of substances to aid in digestion: a. Gastric juice: includes hydrochloric acid, which makes the stomach very acidic, helping to kill bacteria and aid in digestion; pepsin, an enzyme that begins the digestion of proteins; and intrinsic factor, necessary for the absorption of vitamin B12, b. Mucus: secreted by the goblet cells, mucus coats the stomach lining and helps protect it from the acidic gastric juice.
5. *Blood supply*: Branches of the celiac trunk, including the left gastric, the right gastric, the short gastric arteries, and the left gastro-omental (gastroepiploic) artery.
6. *Nerve supply*: By both sympathetic (through the celiac plexus) and parasympathetic nervous systems (primarily through the vagus nerve). Remember to focus on the function of each component part, the role of the stomach in digestion overall, and how different parts can be affected by diseases like peptic ulcer disease or gastric cancer.

### IVS

In which languages can you provide translation from English, with high accuracy in terms of anatomical terminology?

ChatGPT; As an AI developed by OpenAI, I’m capable of translating English text into several languages with a fair degree of accuracy. That includes but isn’t limited to, the following languages: Spanish; French; German; Italian; Portuguese; Dutch; Russian; Chinese; Japanese; Korean; Arabic. It’s important to note, however, that while I strive for the highest accuracy, no translation can be guaranteed to be 100% accurate, especially with complex subjects like medical or anatomical terminology. Certain nuances or specialized vocabulary might not translate perfectly between languages. It’s always a good idea to have important translations verified by a human expert in both the source and target languages.

### IVS

Which of the following is the correct anatomical term? “Sacrotuberous ligament” or “major ischiosacral ligament”?

ChatGPT; The correct anatomical term is “sacrotuberous ligament”, a ligament that runs from the sacrum (lower part of the spine) to the ischial tuberosity in the hip bone. The term “major ischiosacral ligament” is not a recognized term in standard anatomical nomenclature.

### IVS

Please generate a multiple-choice quiz for medical students to test their knowledge of musculoskeletal anatomy

ChatGPT; Sure, here’s a simple multiple-choice quiz about musculoskeletal anatomy:

1. *Which bone is not part of the axial skeleton?* (A) Sternum, (B) clavicle, (C) vertebra, and (D) sacrum
2. *What is the main function of the rotator cuff muscles in the shoulder?* (A) Flexion and extension of the elbow, (B) stabilizing the shoulder joint, (C) adduction and abduction of the wrist, and (D) flexion and extension of the knee
3. *Which of the following is not a carpal bone?* (A) Lunate, (B) capitate, (C) calcaneus, and (D) scaphoid
4. *The linea aspera is a feature found on which bone?* (A) Humerus, (B) tibia, (C) femur, and (D) radius
5. *Which muscle group is primarily responsible for plantar flexion at the ankle?* (A) Quadriceps, (B) hamstrings, (C) gastrocnemius and soleus muscles, and D) gluteals
6. *The bony landmarks known as the greater and lesser tubercles are found on which bone?* (A) Ulna, (B) clavicle, (C) scapula, and (D) humerus
7. *The ‘iliopsoas’ muscle is a composite of which two muscles?* (A) Gluteus maximus and gluteus minimus, (B) psoas major and iliacus, (C) rectus femoris and vastus lateralis, (D) biceps femoris and semitendinosus
8. *Which of the following is not a part of the vertebral column?* (A) Cervical vertebrae, (B) thoracic vertebrae, (C) lumbar vertebrae, (D) coccygeal vertebrae, and (E) cuboidal vertebrae

Replies: 1-B, 2-B, 3-C, 4-C, 5-C, 6-D, 7-B, 8-E.

### IVS

Could you please create another one with increased difficulty?

ChatGPT; Absolutely, I can create a more challenging musculoskeletal anatomy quiz. Here it is:

1. *What is the anatomical term for the socket in the pelvic bone where the femur articulates?* (A) Acetabulum, (B) glenoid cavity, (C) foramen magnum, and (D) fossa ovalis
2. *Which of the following structures is found in the intervertebral disc?* (A) Nucleus pulposus, (B) epiphysial line, (C) medullary cavity, and (D) articular capsule
3. *The structure known as the anatomical neck is found on which bone?* (A) Tibia, (B) humerus, (C) femur, and (D) radius
4. *Which muscles are primarily responsible for pronation of the forearm?* (A) Brachialis and brachioradialis, (B) pronator teres and pronator quadratus, (C) biceps brachii and triceps brachii, (D) supinator and anconeus
5. *Which of the following best describes the anatomical location of the sartorius muscle?* (A) Extends across the back of the scapula, (B) wraps around the lateral side of the upper arm, (C) runs diagonally across the anterior surface of the thigh, and (D) extends along the medial aspect of the lower leg
6. *Which of the following muscles is NOT a part of the quadriceps femoris group?* (A) Vastus intermedius, (B) vastus lateralis, (C) vastus medialis, and (D) biceps femoris
7. *Which ligament of the knee prevents anterior displacement of the tibia relative to the femur?* (A) Anterior cruciate ligament (ACL), (B) posterior cruciate ligament (PCL), (C) medial collateral ligament (MCL), and (D) lateral collateral ligament (LCL)
8. *Which of the following is a primary spinal curvature, present at birth?* (A) Cervical curvature, (B) thoracic curvature, (C) lumbar curvature, (D) sacral curvature

Replies: 1-A, 2-A, 3-B, 4-B, 5-C, 6-D, 7-A, 8-B, 8-D.

### IVS

Please generate a quiz that asks medical students to match different muscles to the corresponding motor nerves

ChatGPT; Sure, I’d be happy to generate a matching quiz for you. Here it is:

1. *Match the muscle to its corresponding motor nerve*: (A) Deltoid, (B) biceps brachii, (C) triceps brachii, (D) gastrocnemius, and (E) quadriceps femoris
2. *Match the motor nerve to its corresponding muscle*: (A) Axillary nerve, (B) musculocutaneous nerve, (C) radial nerve, (D) sciatic nerve, and (E) femoral nerve

Replies: 1-A—> I-axillary nerve, 1-B—> II-musculocutaneous nerve, 1-C—> III-radial nerve, 1-D—> IV-sciatic nerve, and 1-E—> V-femoral nerve.

### IVS

Could you help medical students understand the clinical relevance of an anatomical structure or an anatomical relationship? For example, what is the clinical relevance of the Arcade of Frohse? What is the clinical relevance of the relationship between the radial nerve and the supinator muscle?

ChatGPT; Absolutely. Understanding the clinical relevance of anatomical structures and relationships is crucial in medicine as it provides context and applicability to the material, which can otherwise be quite abstract.

*Arcade of frohse and supinator syndrome:* Also known as the supinator arch, is the most common site of compression for the deep branch of the radial nerve (RN), which innervates the extensor muscles in the posterior forearm compartment. This is clinically significant because compression of the RN here can result in a supinator or radial tunnel syndrome. Patients with supinator syndrome may present with symptoms including pain in the lateral forearm and weakness in the muscles responsible for extending and abducting the hand at the wrist. This can be particularly noticeable when trying to extend the fingers or the wrist against resistance.

*Radial nerve and supinator muscle:* The RN divides into a deep branch, which passes through the supinator muscle, and a superficial branch, which goes on to innervate the skin of the dorsum of the hand. As mentioned above, the deep branch can be compressed at the Arcade of Frohse within the supinator muscle, leading to supinator syndrome. But there’s also another clinically relevant point here. Injuries or surgical procedures involving the area around the elbow or upper forearm must take care to avoid damage to the RN. An injury in this area can result in a ‘wrist drop’, a condition where the patient is unable to extend their wrist and fingers due to paralysis of the muscles innervated by the RN. The patient’s hand may hang flaccidly, and they may be unable to perform tasks such as gripping objects. In all, understanding the anatomy of the RN, including its relationship with the supinator muscle and the Arcade of Frohse, is clinically relevant due to the potential for nerve compression or injury leading to specific motor deficits.

### IVS

Could you provide accurate information about the anatomical variations of a structure? For example, which are the anatomical variations of the AA branching pattern?

ChatGPT; The AA typically gives off three major branches: the STA, the TAA, and the LTA in its first part; the SSA and the ACHA and PCHA in its third part. However, branching pattern variants are not uncommon. Here are a few potential variations:

1. *High origin of the radial artery (RA)*: In some cases, the RA which typically arises from the brachial artery (BA) in the upper arm, can originate from the AA instead,
2. *Aberrant branches*: Additional branches may arise directly from the AA, including the thoracodorsal artery (TDA) or the PCHA,
3. *The variable origin of the SSA*: Sometimes, the SSA can arise jointly with the PCHA or, less frequently, with the ACHA,
4. *Origin of TAA and LTA*: Occasionally, the TAA and LTAs can have a common origin from the AA,
5. *Bifurcation into the radial and ulnar arteries (RA and UA)*: In rare cases, the AA may bifurcate into the RA and UA with no BA present,
6. *Double AAs*: A very rare condition where the AA is duplicated. This usually occurs when the superficial brachial artery (SBA) persists along with the normal deep brachial artery (DBA). These variations are important in surgical procedures involving the axilla, such as mastectomy, axillary lymph node dissection, and shoulder arthroscopy. An awareness of these variations is crucial for surgeons to avoid accidental injury to these vessels during such procedures. They’re also important in diagnostic procedures such as angiography.

### IVS

Which are the anatomical variations of the sciatic nerve (SN) relative to the piriformis muscle (PM)?

ChatGPT; The SN is the largest nerve in the body, and it typically exits the pelvis through the greater sciatic foramen, below the PM. It then descends the back of the thigh to ultimately divide into the tibial and common peroneal (fibular) nerves. However, there are several recognized variants in the relationship of the SN to the PM. These variants can be clinically significant because they may predispose to piriformis syndrome (PS), a condition that can cause buttock and leg pain. Here are the primary variants:

1. *Type I (most common)*: The SN emerges below the PM, as a single nerve (84% of people),
2. *Type II*: The SN pierces through PM (7.7% of people),
3. *Type III*: The common peroneal portion of the SN passes through PM, while the tibial portion passes below (0.5%),
4. *Type IV*: The common peroneal portion passes over the PM, while the tibial portion passes below (1.2%),
5. *Type V*: The common peroneal portion passes through PM, while the tibial portion passes over it (0.6%),
6. *Type VI*: The SN divides into the tibial and common peroneal nerves in the pelvis. The common peroneal nerve passes over the PM and the tibial nerve passes below (4.3%). These variants may contribute to a PS, in which the SN is compressed or irritated by the PM. This can cause pain, tingling, or numbness in the buttocks, and along the path of the SN descending the lower thigh and into the leg. Awareness of these variants is essential in accurately diagnosing and treating PS.

### IVS

As a teacher, I’d like to stress to the students the importance of anatomy in ME. Could you help me with that?

ChatGPT; Absolutely. Understanding anatomy is foundational to the practice of medicine. Here are some reasons to stress the importance of anatomy in ME:

1. *Understanding disease process*: To understand disease, you must first understand normal structure and function. Anatomical knowledge enables doctors to understand the location of disease, its development, progression, and its implications on the body,
2. *Patient examination*: Physical examination skills are built on a solid understanding of anatomy. From basic skills like palpation, auscultation, and percussion, to more advanced skills like neurological and orthopedic examination, anatomy underpins them all,
3. *Medical procedures*: Nearly all medical procedures, from a simple blood draw to advanced surgical procedures, require a good understanding of anatomy. For instance, surgeons need to know the exact location of organs, blood vessels, nerves, and other structures to perform operations safely and effectively,
4. *Interpretation of diagnostic tests*: Reading and interpreting X-rays, CT scans, MRIs, and other diagnostic tests require a thorough knowledge of anatomy. Radiologists, need to understand the 3D relationships of structures to interpret these images,
5. *Communication*: Precise anatomical knowledge allows for clear, unambiguous communication among healthcare professionals. It provides a universally accepted language to describe the body and its conditions, ensuring patient safety and effective treatment,
6. *Pharmacology*: Understanding where in the body a drug will act, how it will get there, and what effect it will have on tissues and organs, all require an understanding of anatomy,
7. *Patient education*: Doctors often need to explain medical conditions and treatments to patients. Being able to describe these in the context of the body's anatomy can make these discussions more understandable to patients. Remember, anatomy is not just a pre-clinical subject. Its principles should be applied throughout medical training and beyond. While the sheer volume of information can be daunting, understanding and remembering that every piece of anatomical knowledge has a potential application will make the process more rewarding and interesting.

### IVS

Could you pick a highly cited article from surgical and radiologic anatomy and provide a summary for a medical student?

ChatGPT; I’m sorry for the misunderstanding, but as of my last update in September 2021, I don’t have the ability to access or search the internet in real-time, including specific databases or journals. Therefore, I can’t pull up or summarize specific articles, whether from Surgical and Radiologic Anatomy or other sources. However, I can demonstrate how to summarize a scientific article using a fictitious example. Here’s an illustration of how a summary for a medical student might look: *Title*: “Anatomical Variations in the Sural Nerve and Its Implications in Surgery: a systematic review” *Summary*: This review paper aimed to consolidate current knowledge on the anatomical variations of the sural nerve and discuss its surgical implications. The sural nerve, a sensory nerve in the calf, is commonly used in nerve grafting procedures, and understanding its anatomy is crucial for successful surgical outcomes. The authors examined numerous studies on the subject. They found that the sural nerve’s course, branching pattern, and connection with the lower lateral cutaneous nerve of the calf and the peroneal communicating nerve could vary significantly among individuals. Specifically, the study discussed how these variations could affect nerve grafting procedures and potentially lead to postoperative sensory deficits in the donor area. They stressed the importance of using imaging techniques such as ultrasound or MRI to identify the nerve’s course and any potential variations preoperatively. The authors concluded that a deeper understanding of these anatomical variations and preoperative planning could help optimize surgical outcomes and minimize patient discomfort post-operation. Remember that when reading a scientific paper, it’s important to understand the objective, methodology, key findings, and conclusion of the study. Critical appraisal of the study’s validity and relevance is also a key skill in evidence-based medicine.

### IVS

Can you provide a summary of the article entitled “Ulnar trochlear notch articular surface has three morphological patterns: a neglected major anatomical feature”, which was published in 2019 in the surgical and radiologic anatomy journal [3], for a medical student?

ChatGPT; I’m sorry for any confusion, but as an AI model developed by OpenAI, I don’t have direct access to my training data or know where it comes from. However, I was trained on a mixture of licensed data, data created by human trainers, and publicly available data. I have not been directly trained on specific databases or have the ability to access or retrieve personal data unless it has been shared with me in the course of our conversation. I am designed to respect user privacy and confidentiality. My primary function is to provide information and answer questions to the best of my knowledge and abilities. If you have any concerns about privacy or data security, feel free to let me know, and I will do my best to address them. Unfortunately, I don’t have the ability to access databases, subscription sites, confidential information, or proprietary resources, so I can’t provide a summary of the specific article from "Surgical and Radiologic Anatomy" you mentioned. I don’t have the ability to access the internet, retrieve, or know specific documents or sources. I recommend reading the abstract for a summary of the paper. The introduction will give you background information and the context of the study, the methods section will tell you how the study was conducted, the results section will present the findings, and the discussion and conclusion sections will interpret the results and suggest further research. I can help guide you on how to approach reading scientific articles.

### IVS

Below is the full text of the article in question. Can you provide a summary to a medical student? (the main text of the article was copied and pasted to the question)

ChatGPT; This study, titled “Ulnar trochlear notch articular surface has three morphological patterns: a neglected major anatomical feature”, is centered around the morphology of the ulnar trochlear notch and the articular surface variations found in dried ulnae. The ulnar trochlear notch is a part of the ulna that articulates with the trochlea of the humerus. The typical anatomy involves a non-articular, rough transverse ridge, described as the "bare area," separating the olecranon and coronoid facets. However, this research uncovered that this typical anatomical pattern is not as common as traditionally believed. Out of 273 unpaired ulnae, 165 (60.4%) exhibited the traditional separate facets (type I), but a substantial percentage (39.6%) showed variations, where the olecranon and coronoid facets were either partially fused (type II—27.5%) or completely fused (type III—12.1%). This morphology differed between genders, with type II and III being more common in women than the traditional separate facets (type I). The authors highlighted the clinical implications of these anatomical variations, particularly in surgical procedures like olecranon osteotomies and elbow arthroscopy, where a failure to recognize these variations could potentially lead to iatrogenic injury. For instance, in elbows without a transverse ridge (type III), which represent about 40% of the cases, an olecranon osteotomy might damage the articular cartilage, potentially causing secondary osteoarthritis. In addition to surgery, these variations might also affect interpretations of imaging studies, as the absence or presence of the transverse ridge could be mistaken for a fracture or an intraarticular osteophyte on CT or MRI scans. The study concludes that while the traditional anatomical pattern with separate facets is most common, it’s crucial to recognize and understand these variations in the ulnar trochlear notch’s articular surface. This recognition is vital not only for the accurate interpretation of imaging studies but also for surgical planning and avoiding iatrogenic injuries.

### IVS

Αnatomical illustrations are a cornerstone in anatomy teaching. Can you provide any anatomical illustrations if requested?

*ChatGPT*; As a text-based AI developed by OpenAI, I’m unable to create or provide visual content such as anatomical illustrations or diagrams. However, I can describe anatomical structures in detail, provide textual explanations of anatomical relationships, and guide you to appropriate resources or textbooks for these illustrations. If you’re looking for a specific illustration or want to understand a particular structure better, I suggest referring to reputable anatomy textbooks or online resources such as xxxxx, Atlas of xxxxx by xxxxx, or digital platforms like xxxxx or xxxxx (blinded to avoid advertising). These resources provide high-quality, accurate anatomical illustrations. I recommend using these visual resources in conjunction with our discussions to maximize your understanding of human anatomy.

### IVS

Could you please critique and suggest any ways to improvement of your capabilities in helping medical students with AE?

ChatGPT; As an AI, I strive to provide as accurate and useful information as possible, and I’m constantly being updated and improved based on feedback and advancements in technology. Here are some areas where there may be room for enhancement when it comes to supporting medical students in their AE:

1. *Visual aids*: While I can provide detailed descriptions of anatomical structures and processes, I currently lack the capability to provide visual aids like diagrams or sketches. Visual aids are critical in learning anatomy, so the ability to generate accurate anatomical diagrams could be extremely beneficial.
2. *Practical applications*: It would be helpful if I could offer more interactive experiences, such as virtual dissections or 3D modeling of anatomical structures. This could help bridge the gap between theoretical knowledge and practical, hands-on experience,
3. *Personalization*: Tailoring the information to the individual needs of each student could greatly enhance the learning experience. For instance, if I could understand a student’s strengths, weaknesses, and preferred learning styles, I could provide more customized advice and resources,
4. *Real-time updates*: While my training includes extensive medical and scientific information, it is current only up until September 2021, and I can’t access real-time data or updates. This limitation could be addressed by allowing me to access the latest research and medical guidelines,
5. *Interactive learning*: Implementing more interactive forms of learning, such as quizzes or flashcards, could make studying more engaging and effective for students,
6. *Communication skills*: While I strive to provide accurate information, sometimes my replies can be quite technical and difficult for non-experts to understand. Improvements could be made to ensure that I communicate complex information in an easy-to-understand manner,
7. *In-depth critique and analyze*: While I can summarize and explain content from medical papers and textbooks, my ability to critically analyze or provide an opinion on these is limited as I don’t possess subjective experiences or beliefs. Please note that while these improvements could help make AI more effective for AE, they should be designed and implemented with careful consideration of ethical implications, such as data privacy and the risk of misinformation.

### IVS

Thanks. We hope that our conversation may be helpful to improve the engagement of medical students in studying anatomy

ChatGPT; Absolutely! Conversations like ours are incredibly valuable for understanding how to better leverage AI technology in education, especially in complex and critical fields like anatomy. Medical students have a challenging journey, and every tool that can make their learning more efficient and engaging is worth exploring. As advancements continue, AI can be improved and better integrated into the learning process to assist students in a more personalized and interactive way. Please feel free to ask if you have any more questions or need further assistance in the future. Your insights and inquiries are always appreciated.

## Discussion—commentary

In the beginning, the chatbot acknowledges that it may not always provide accurate replies and is meant to be an AE supplement. When discussing how generative language models can assist medical students in AE, ChatGPT mentions interactive learning tools like simulated doctor-patient scenarios. While these can be useful for ME, their relevance to AE may be limited as anatomy is typically taught early on, before students have gained experience in patient interaction, diagnostic thinking, and reviewing patient records. It seems the chatbot is capable of providing concise chapter summaries in bullet point format, which can aid students in their studies. It could be beneficial for a student to have the option of creating multiple-choice quizzes or matching questions with different levels of difficulty while preparing for an exam. When asked about anatomical features, this source provides detailed and well-organized responses, recognizing the existence of variants and offering prompts to connect specific features to their functions and clinical implications. However, it was noted that certain details were omitted, such as the role of the right gastro-omental (gastroepiploic) artery in supplying blood to the stomach and the drainage of veins from the stomach. We noticed some mistakes in the information provided, such as the location of the pancreas. It is actually located in a posterior-inferior position, rather than solely inferior to the stomach. The ChatGPT can offer helpful advice on anatomical terminology. While it may not provide as much information on variants compared to other sources, it does highlight their clinical impact. When a variant is classified into types, the ChatGPT shows higher precision with minor mistakes in its replies. Although previously correctly analyzed the AA branching pattern, a question about the AA variant branching pattern failed to identify the 2nd part branches (thoracoacromial and lateral thoracic artery origin). In questions asked by Mogali, we noticed more noticeable omissions and inaccuracies [2]. In the current study, the updated version of ChatGPT was utilized which is believed to be better than its previous versions. However, the accuracy of the replies provided may vary depending on the nature of the question asked. ChatGPT can be a useful tool for medical students to comprehend the clinical significance of anatomical structures and their adjacent areas. Although it provided a thorough explanation of the clinical relevance of the relationship between the radial nerve and supinator muscle, an anatomy professor may emphasize the crucial role of pronation and supination in this relationship, especially for surgical procedures in the area.

ChatGPT-4 cannot conduct real-time searches for articles, even if the author’s information is provided. This was highlighted in two previous articles [1, 2], where the chatbot provided summaries of fake articles, which received criticism in the comments. However, if the user provides the chatbot with the full text of an article, it can then provide a summary. ChatGPT-4 emphasizes the importance of learning anatomy from various perspectives to promote a thorough understanding of the subject matter and its value.

### ChatGPT and the study’s limitations

A notable drawback of the chatbot is that it cannot provide images. Instead, it directs users to anatomy textbooks and digital platforms for illustrations. This is a significant limitation as images are crucial for understanding anatomy. Additionally, since the chatbot does not provide citations in its replies, its statements cannot be considered evidence-based. Another limitation is that the chatbot, ChatGPT-4, lacks knowledge of research data published after its last update in September 2021. Despite these limitations, ChatGPT-4 acknowledges its weaknesses and recognizes the potential for improvement. It is important to note that the present study only explored limited anatomical topics to assess the chatbot’s potential impact on ME and MR. Therefore, the accuracy of the chatbot’s replies may vary if users ask about different anatomical structures. However, the study’s objective was to provide insight into the chatbot’s current capabilities. Furthermore, the chatbot’s database is continuously updated, which retrains the model and may lead to subtle changes in its responses over time.

## Conclusion

Based on the recent interview, it appears that ChatGPT-4 could be a valuable interactive anatomy tool for students. This chatbot has the potential to increase engagement by encouraging students to ask further questions. It provides detailed and organized descriptions of anatomy, concise chapter summaries in bullet point format, and accurate advice on anatomical terminology. The chatbot also highlights the clinical significance of variants, but its replies are limited unless a variant is classified into types. Additionally, it helps medical students comprehend complex anatomical terms and understand the clinical relevance of a structure and its relationship. ChatGPT-4 can generate quizzes of varying difficulty levels, including multiple-choice and matching questions, and can summarize articles when presented with the text. It’s important to note that while ChatGPT-4 cannot replace an anatomy teacher, it recognizes that AI should complement the important role of human educators. Future research could provide guidelines for the optimal use of ChatGPT-4 in ME.

## Data Availability

The original copy of the interview is available.
